# An Anatomic Reduction Technique for Salter-Harris Type III Medial Femoral Condyle Fractures

**DOI:** 10.1016/j.jposna.2026.100422

**Published:** 2026-07-08

**Authors:** Aaron Tran, Stephanie T. Kha, Christine L. Farnsworth, Eric W. Edmonds, Christopher D. Souder

**Affiliations:** 1 Department of Orthopaedic Surgery, University of California San Diego Health, San Diego, CA, USA; 2 Division of Orthopedics, Rady Children's Hospital, San Diego, CA, USA

**Keywords:** Salter-Harris III, Salter-Harris 3, Medial distal femoral condyle fracture, Distal femoral physeal fracture

## Abstract

Salter-Harris type III (SH-III) distal femur fractures are challenging to manage given their intra-articular nature and physeal involvement. Inadequate reduction of these injuries can potentiate growth disturbances, including premature physeal closure and subsequent deformity. Existing literature lacks a description of a standardized reduction technique for this fracture pattern. In this report, we use a cadaver knee to demonstrate a structured reduction maneuver for the SH-III medial femoral condyle fracture, designed to facilitate anatomic alignment of the epiphyseal fragment via closed or minimally invasive means. The technique involves manipulating the fracture with the knee in a gently flexed position. In this position, the lower leg is externally rotated while a varus force is applied to the knee. This allows the medial meniscus to aid in reducing the medial femoral condyle into its anatomic position. We also present clinical cases demonstrating the in vivo success of the maneuver in achieving reduction of SH-III medial femoral condyle fractures. This technique strategically uses the local anatomy to achieve anatomic reduction without the need for aggressive manipulation.

**Key Concepts:**

(1) Anatomic reduction of Salter-Harris type III distal femoral fractures is critical to preserving physeal integrity and joint congruity.
(2) An external rotation maneuver of the leg, while applying a varus force to the knee, can reproducibly restore alignment in a Salter-Harris type III medial femoral condyle fracture.
(3) The technique exploits the juxtaposition of the medial meniscus to aid in condylar reduction.
(4) The reduction method may allow percutaneous fixation with minimal intraoperative soft-tissue dissection and may reduce the need for extensive open reduction.

## Introduction

Salter-Harris type III (SH-III) fractures of the distal femur most commonly occur in adolescents after sports-related trauma or motor vehicle collisions. Injury typically results from hyperextension and/or varus or valgus stress to the knee, leading to epiphyseal separation because the physis has lower tensile strength than the adjacent ligaments [1,2]. The most common SH-III pattern is a valgus injury with stress along the medial collateral ligament, resulting in a fracture of the medial femoral condyle [3]. Although less common than SH-II fractures, SH-III fractures are clinically significant physeal injuries in the pediatric population because of their intra-articular nature and physeal involvement. The distal femoral physis accounts for approximately 70% of femoral longitudinal growth and 40% of total lower-extremity limb length [4,5]. Therefore, even minor disruptions can have profound consequences, including limb length discrepancy, angular deformities, and joint incongruity that may lead to posttraumatic arthritis [2,[6], [7], [8]]. Up to 22% of patients with a distal femoral physeal fracture may experience a leg length discrepancy of 1.5 cm or greater [4]. Fractures with any degree of displacement are four times as likely to result in growth arrest as nondisplaced fractures. Notably, the rate of growth disturbance increases with Salter-Harris grade, with approximately 49% of SH-III fractures resulting in growth arrest [4]. The nature of the injury and its associated complications underscore the importance of achieving an anatomic reduction to minimize this risk [3,7,8].

Closed reduction techniques can fail due to deforming forces and/or periosteal entrapment. Moreover, rotational and translational malalignment can be subtle and difficult to correct with simple traction or gross manipulation. Repeated attempts at reduction may lead to iatrogenic injury to the physis. This report describes an anatomic reduction maneuver for SH-III medial femoral condyle fractures, using lower-leg external rotation and a varus knee force while the knee is in gentle flexion. Using a human donor knee, we characterize the soft tissues that can be used to allow this manual maneuver to consistently restore anatomic alignment. Additionally, we provide clinical examples of the practical application of the technique in the pediatric population, using both an open approach and an arthroscopic approach to fracture management.

## Description of the method

### Human donor knee model preparation

**Figure 1:**
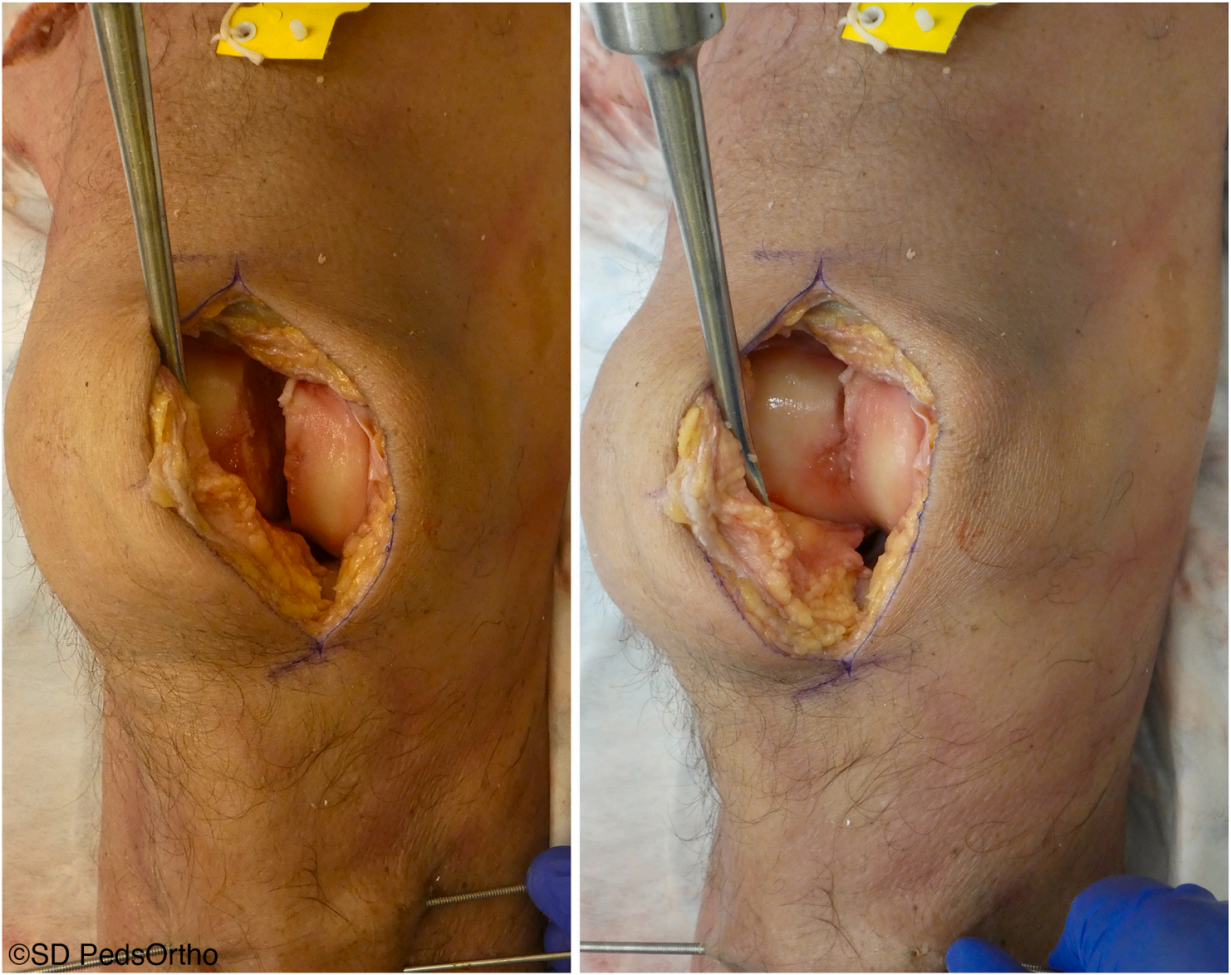
Adult donor knee clinical images. (Left) Displaced simulated medial femoral condyle fracture. (Right) Anatomic reduction of the fracture while external rotation and a varus force are being applied.

**Figure 2:**
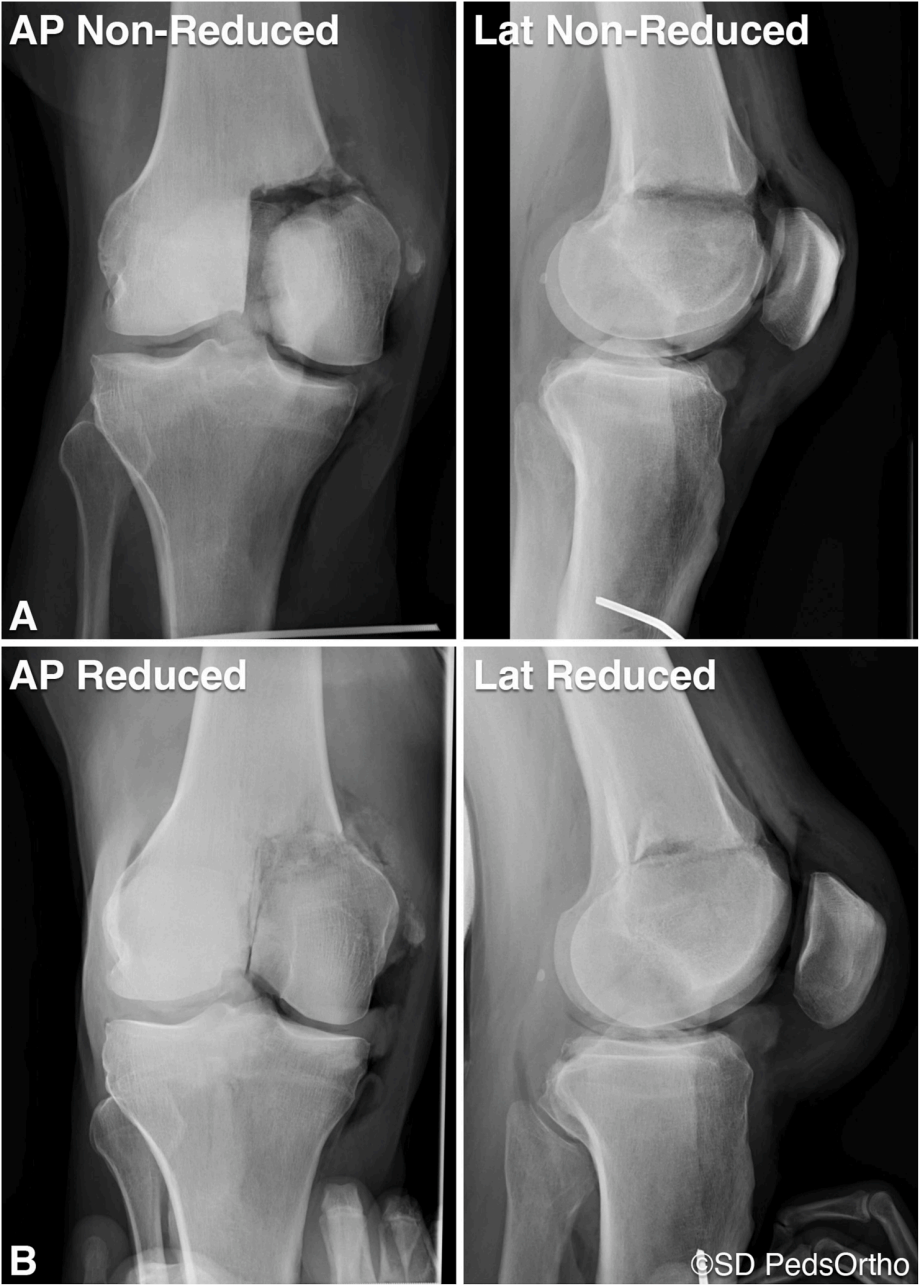
Radiographic images of (A) SH-III fracture and (B) post-reduction images. *SH-III, Salter-Harris type III.*

A fresh-frozen donor right knee (54-year-old male, body mass index: 23.01) was thawed overnight before an SH-III fracture was simulated in the distal medial femur. An osteotome and mallet were used to create a medial condylar osteotomy to reproduce the proposed fracture pattern (Fig. 1). The procedure was performed by a pediatric orthopaedic fellowship-trained surgeon. Prereduction radiographic imaging confirmed the SH-III fracture morphology (Fig. 2A).

Next, a limited medial parapatellar arthrotomy was performed to expose the distal femur while preserving key soft tissue attachments for assessment of their contributions to the reduction of the epiphyseal fragment. The reduction technique was performed under direct visualization and radiographic imaging. Direct observation of the reduction maneuver allowed determination that the medial meniscus and medial capsule are most responsible for returning the condyle into anatomic position (Fig. 2B). With external rotation, these structures provided a buttressing effect, producing the reduction (See Donor Knee video in Additional Links section).

## Reduction technique

### Patient positioning

The knee is positioned supine on a radiolucent table with a small bump to allow unrestricted biplanar fluoroscopy access.

### Reduction sequence

1. **Flexion (20-30°):** Reduces posterior capsular tension, increasing epiphyseal mobility. Particularly for posteriorly displaced fragments, this allows for anterior translation of the displaced fragment.
2. **Varus Stress:** Directs the epiphyseal fragment back laterally toward the anatomic position and counters typical medial displacement patterns. The tibia and medial meniscus provide a buttress effect on the epiphyseal fragment to aid in reduction.
3. **External Rotation:** External rotation of the lower leg allows the medial meniscus and medial capsule to guide the medial femoral condyle into its proper position. ***External rotation is the key maneuver for realigning the articular surface.***
4. Once reduction is determined to be satisfactory, definitive stabilization can then be performed by inserting percutaneous lag screws while maintaining physeal preservation principles.

### Case series

#### Open approach

**Figure 3:**
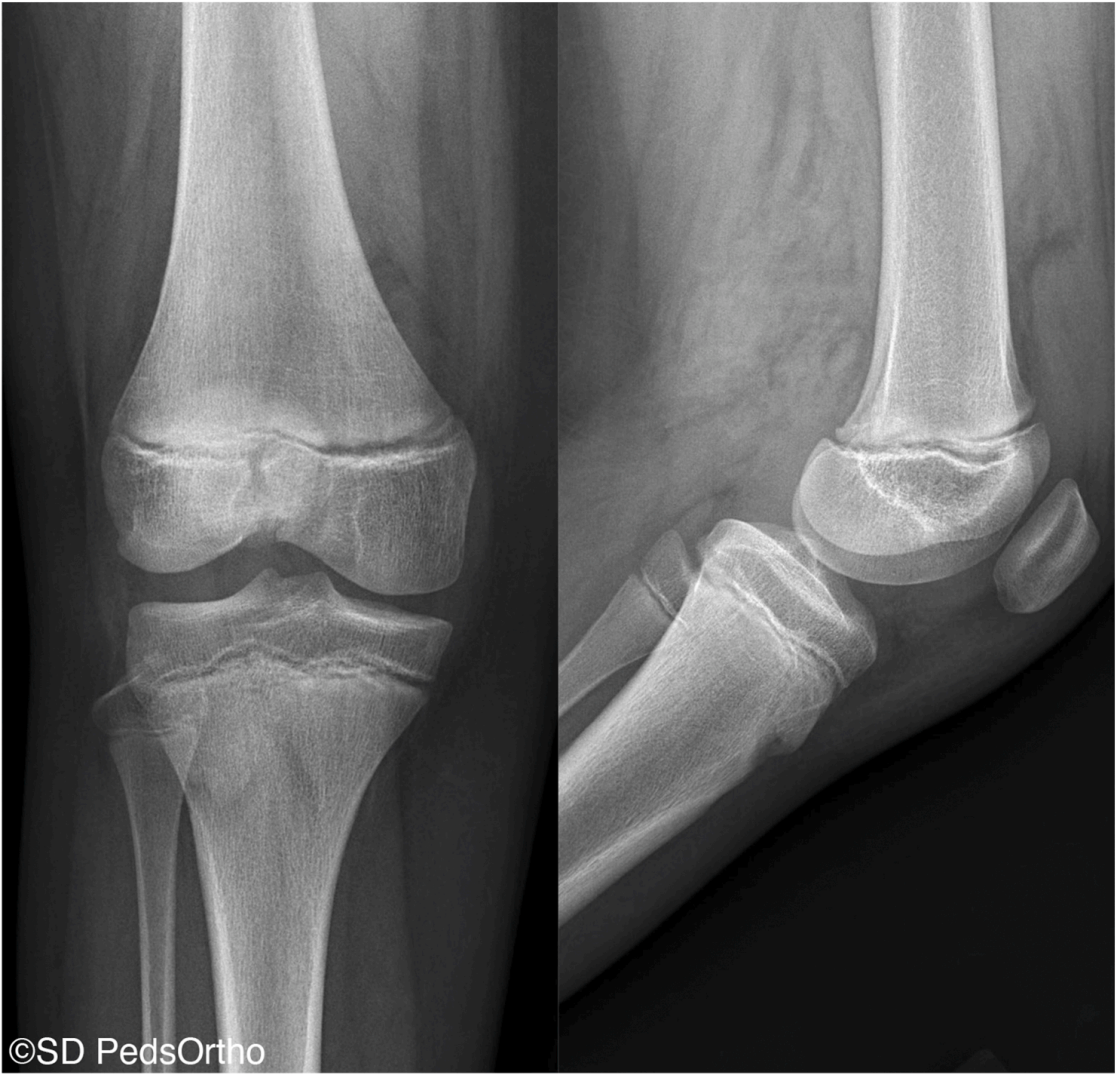
Radiograph of a right knee injury in a 10-year-old female.

**Figure 4:**
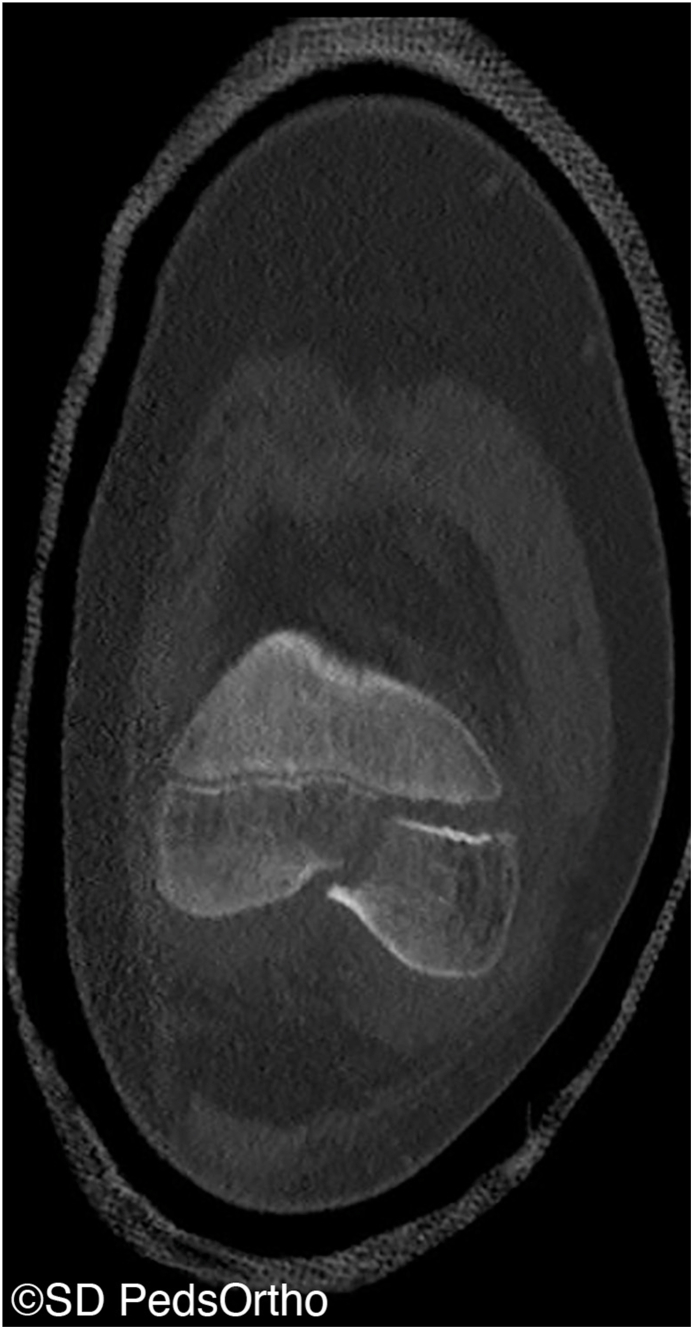
Coronal view of the distal femur Salter-Harris type III fracture from CT imaging. *CT, computed tomography.*

**Figure 5:**
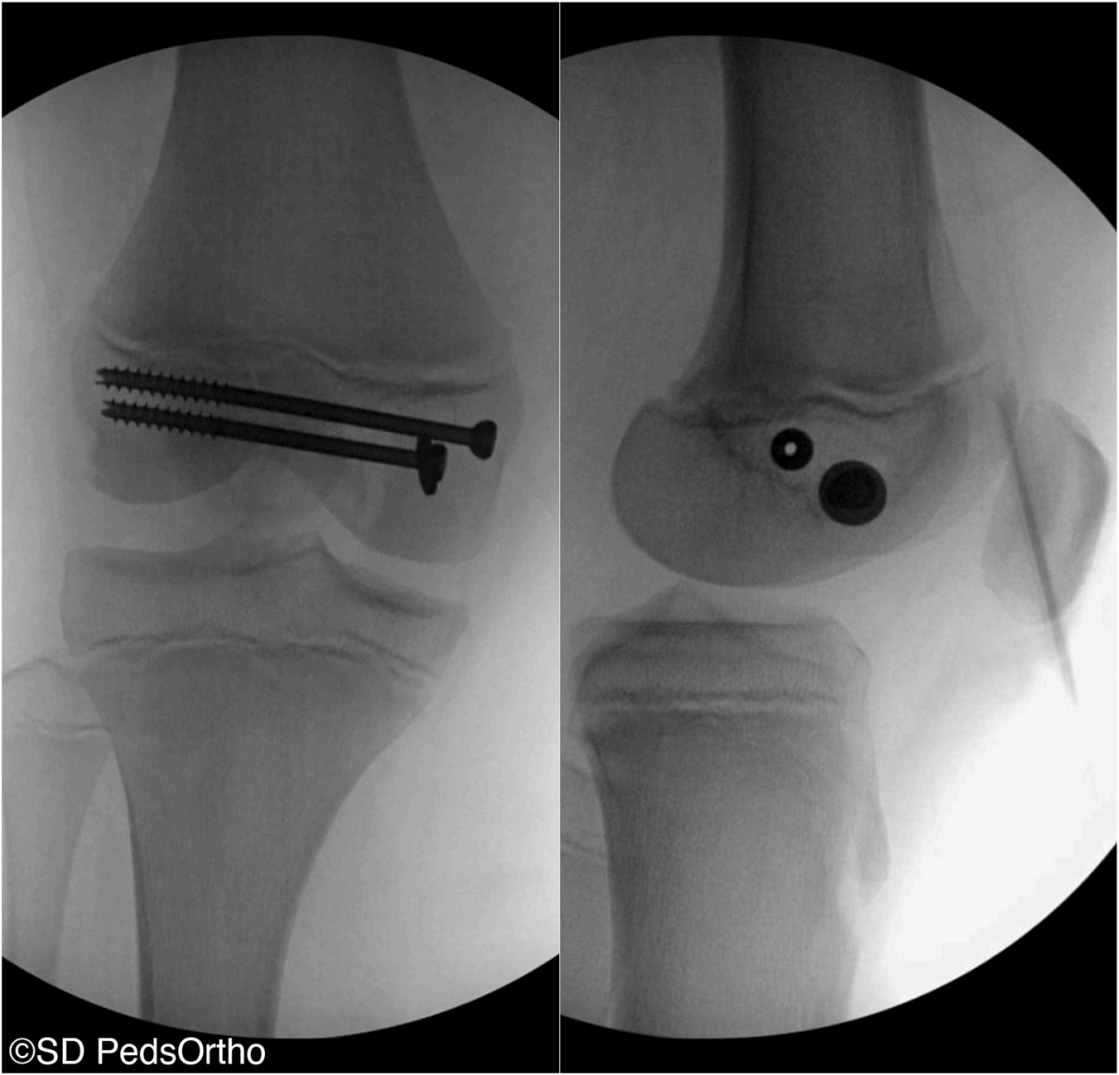
Fluoroscopy image taken interoperatively.

**Figure 6:**
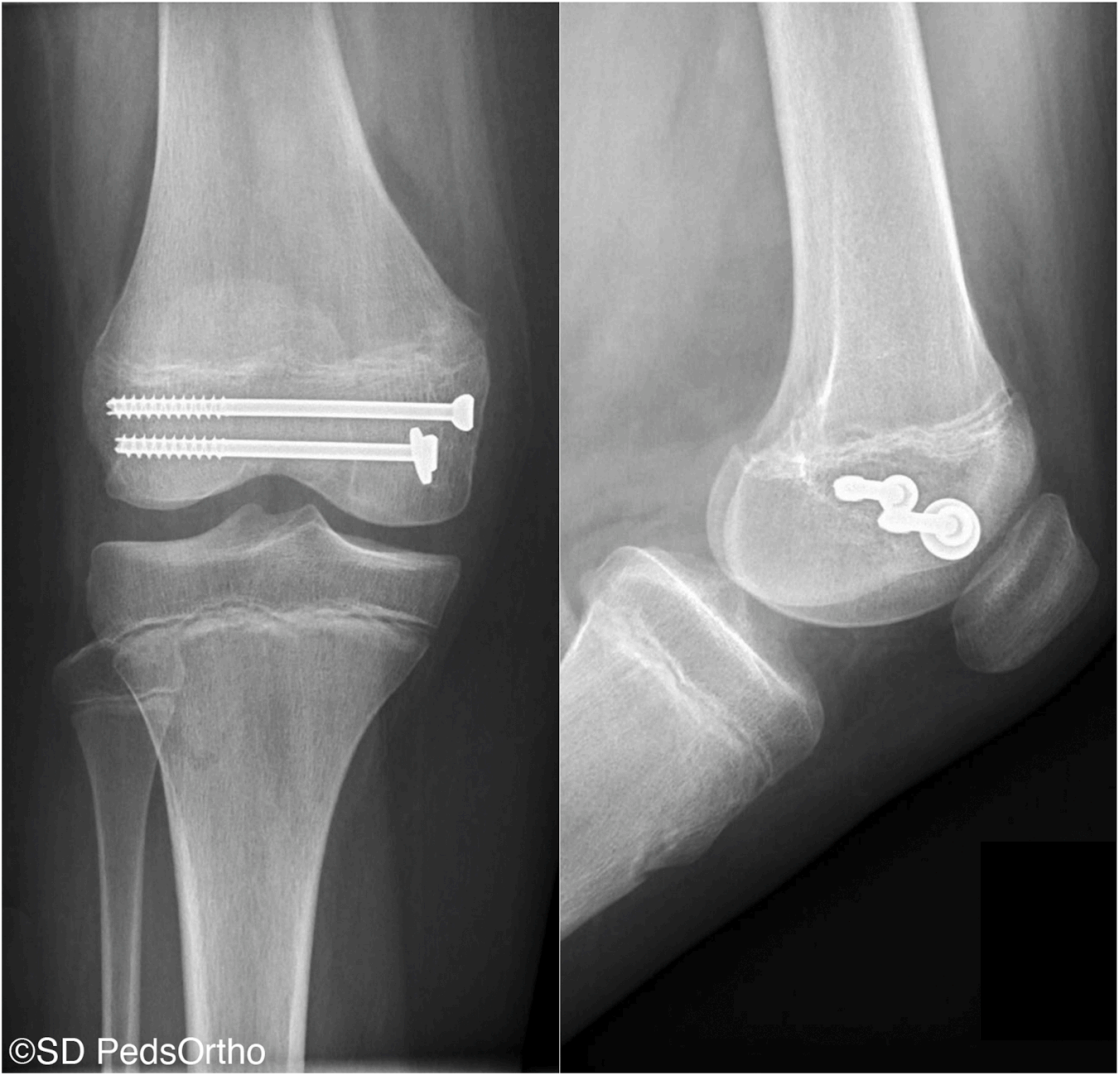
Radiograph taken three months postoperatively. Her distal femoral physis appears somewhat narrowed, and she will be closely followed to determine if she develops angular deformity or leg length discrepancy.

Case 1: A 10-year-old female sustained a right knee injury in a bounce house. She had immediate right knee pain and was unable to bear weight. She was brought to the Emergency Department, where radiographs revealed a distal femoral physeal fracture (Fig. 3). A CT scan was performed and demonstrated a displaced SH-III medial femoral condyle fracture (Fig. 4). She was taken to the operating room for open reduction and internal fixation. A subvastus approach was used to visualize the fracture and the reduction. After gentle debridement of the hematoma, the above-described reduction maneuver was performed, producing an anatomic reduction of the joint surface (see Open Approach Case video in the Additional Links Section). A clamp was used to maintain the reduction, and two partially threaded screws were used to stabilize the fracture (Fig. 5). The patient was allowed early range of motion but no weight-bearing for 4 weeks. Three-month follow-up demonstrated healing of the fracture and anatomic alignment of the joint surface (Fig. 6). As this patient is at risk for physeal bar formation with femoral shortening, she requires careful long-term follow-up.

#### Arthroscopic approach

**Figure 7:**
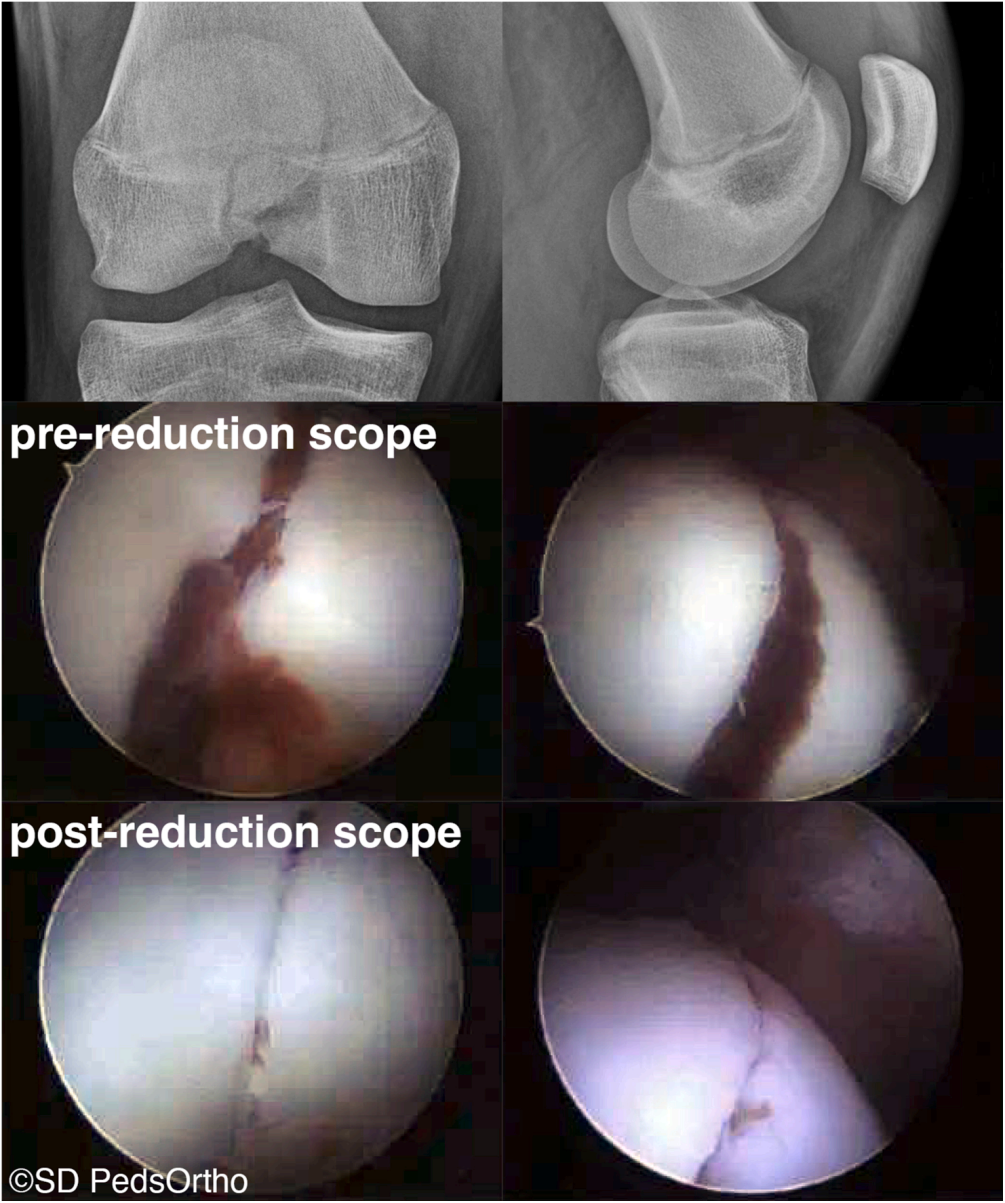
Injury radiographs from 16-year-old male. Arthroscopic images showing the fracture before reduction (top row, left near the notch and right across the condyle) and images following the presented reduction maneuver (bottom row representing the juxtaposed top row image).

**Figure 8:**
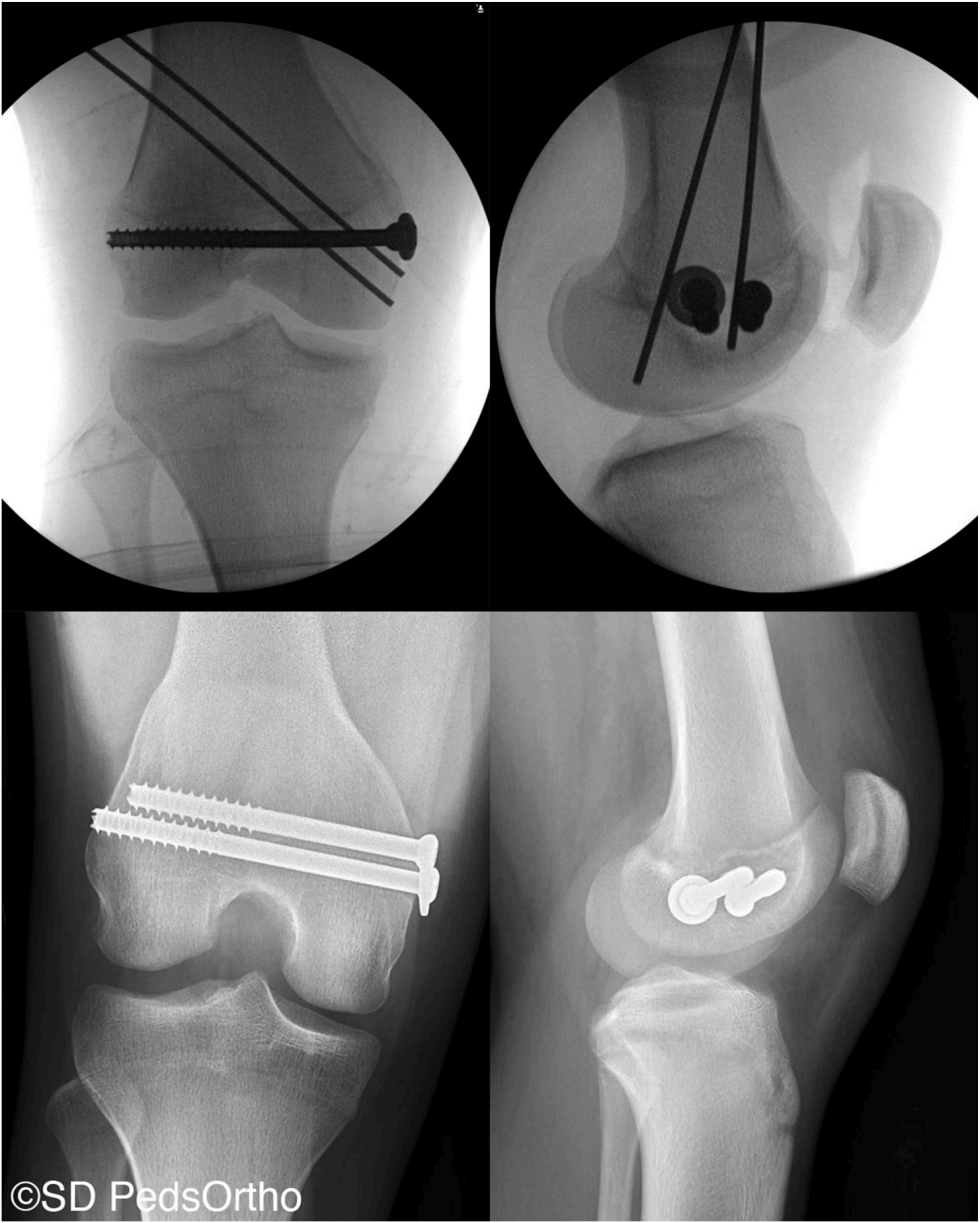
Intraoperative fluoroscopy (top) and postoperative radiographs (bottom).

**Figure 9:**
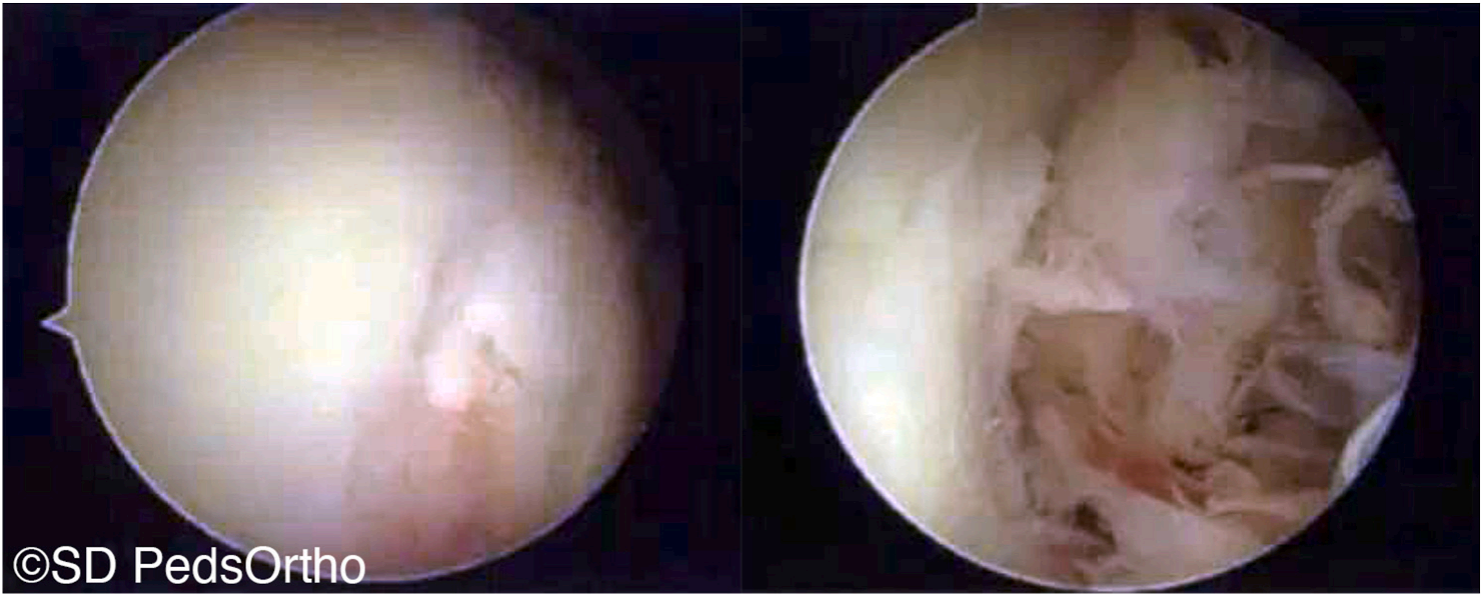
Arthroscopic images 3 months after index procedure demonstrating maintained reduction and articular surface congruity at the fracture site (left image) and arthrofibrosis at the notch that resulted in knee stiffness, which underwent a subsequent lysis of adhesions.

Case 2: A 16-year-old male presented with a right knee injury after being struck in the knee by another player during a baseball game. An outside ED obtained radiographs and an MRI, identifying an SH-III medial distal femoral condyle fracture. Surgery was performed 3 days later. Standard anterior parapatellar portals were used for diagnostic arthroscopy of both the medial. and lateral compartments under tourniquet inflation. An oscillating shaver was then used to perform minor debridement of the ligamentum mucosum and anterior fat pad to improve visualization, as well as excision of the easily visible hematoma around the fracture site. The cruciate ligaments and menisci appeared intact. Next, the described reduction maneuver was performed to reduce the fracture, which was then cross-clamped to tentatively hold the reduction. The articular surface appeared reduced (Fig. 7), and fluoroscopy was used to confirm the fracture reduction. Two 6.5 mm cannulated screw sets were used to secure the fracture to the lateral condyle. Two 2.0 mm pins were then placed from the distal medial fragment to the proximal lateral metaphysis. These pins were driven out of the skin and laterally to leave a minimal amount of pin in the joint while remaining bicortical (Fig. 8). The patient was placed in a cast for 3 weeks. At 3 months postoperatively, the knee had only 0–95 degrees of range of motion, so the patient returned to the operating room for an arthroscopic lysis of adhesions, regaining full unrestricted motion (Fig. 9). Return to sport occurred at 8 months postoperatively.

**Figure 10:**
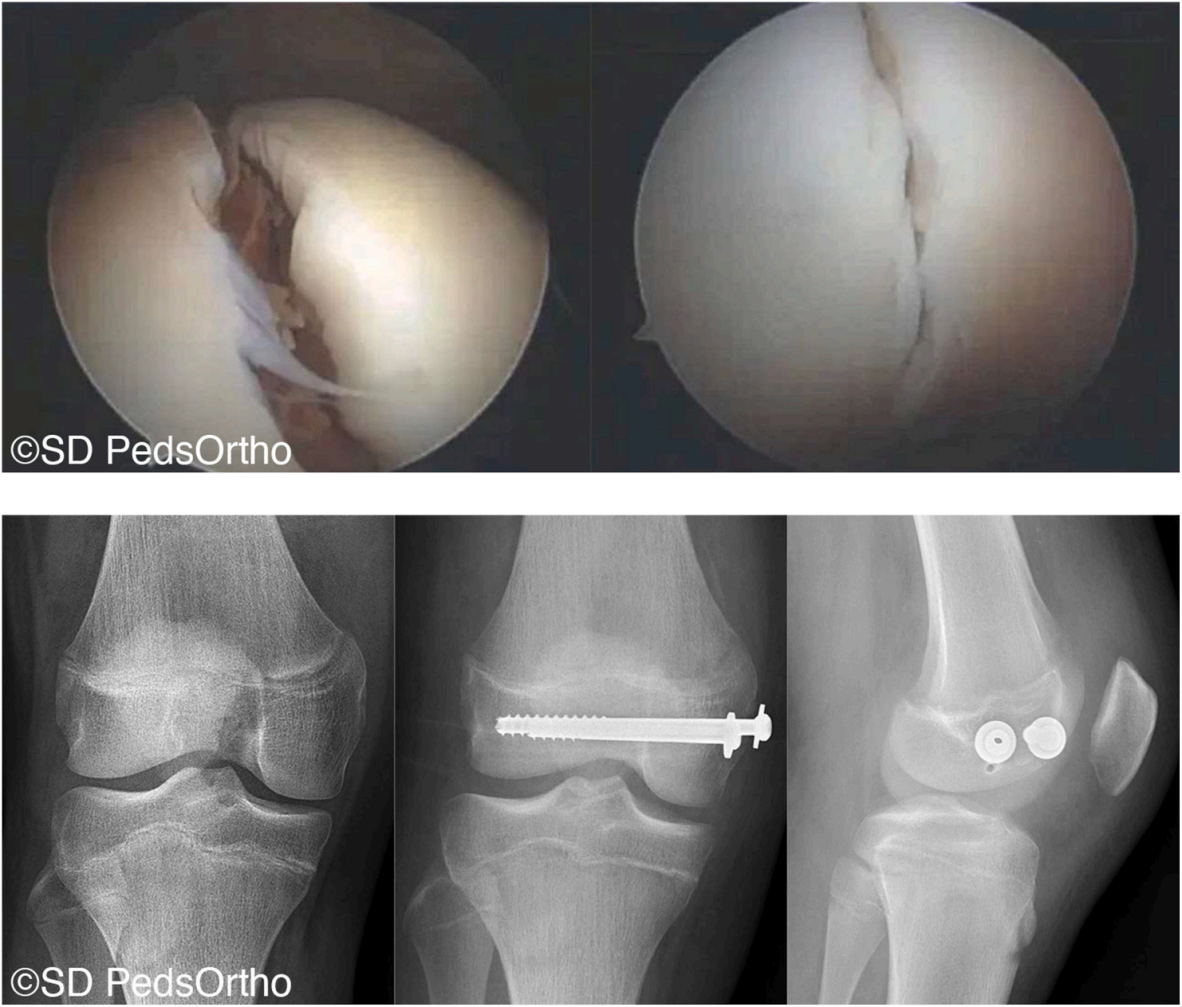
TOP: Preoperative (left) and postoperative arthroscopic images (right). BOTTOM: Preoperative (left) and postoperative radiographs.

**Figure 11:**
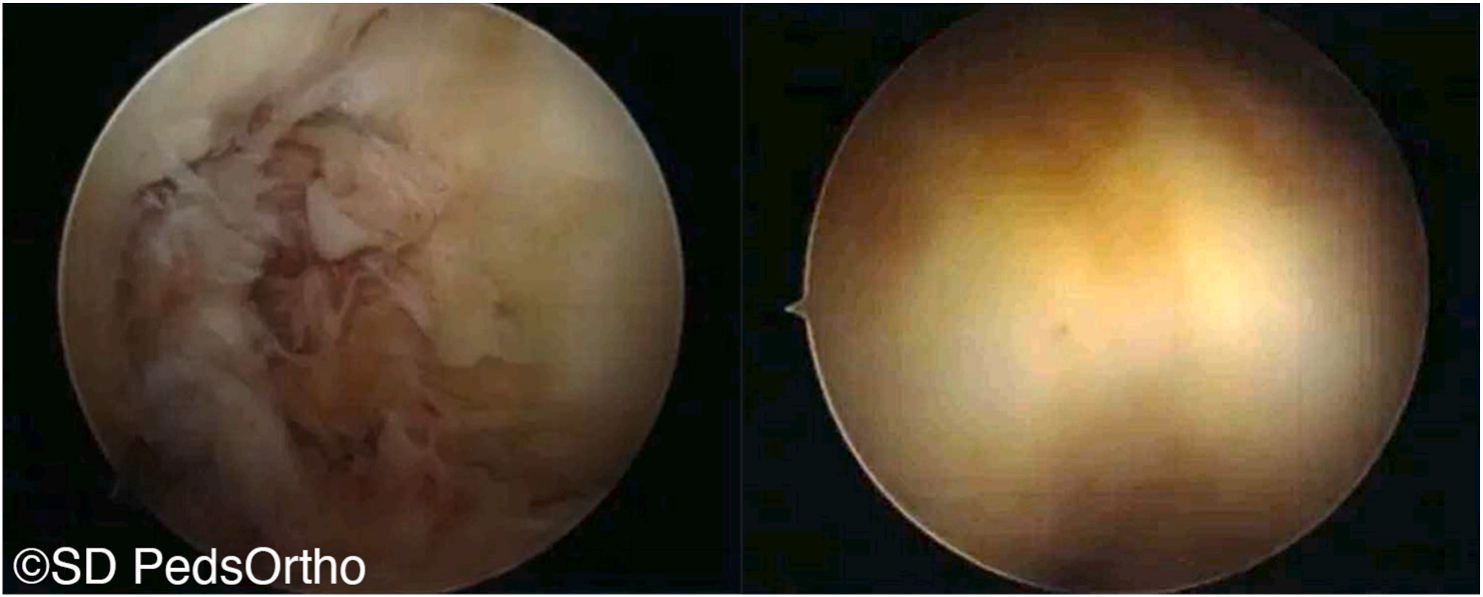
Four months following injury, anterior arthrofibrosis (left image) that underwent lysis of adhesions and demonstrated maintenance of joint congruity above the notch at the healed fracture.

Case 3: A 14-year-old male presented to the clinic after sustaining a right knee injury when he was "struck from both sides" of his knee. Radiographs revealed an SH-III distal femur fracture. The patient underwent surgery 8 days after the injury using an arthroscopic approach. The reduction was obtained with external rotation and a varus maneuver, and the fracture was repaired with 6.5 mm cannulated screws (Fig. 10). A knee immobilizer was used during the postsurgical period and was discontinued at 1 week, when he was converted to a knee range-of-motion brace and formal physical therapy was initiated to work on range of motion, with no weight-bearing for the first 4 weeks. He slipped and fell, catching himself with the operative leg 3 weeks postoperatively, resulting in significant pain. At 6 weeks postoperatively, he was able to achieve only 0–55 degrees of knee motion. Therefore, at 4 months postoperatively, he underwent arthroscopic lysis of adhesions, which restored symmetric knee range of motion after removing significant anterior arthrofibrosis and adhesions to the cartilage and fracture itself (Fig. 11). He returned to playing football the following season.

**Figure 12:**
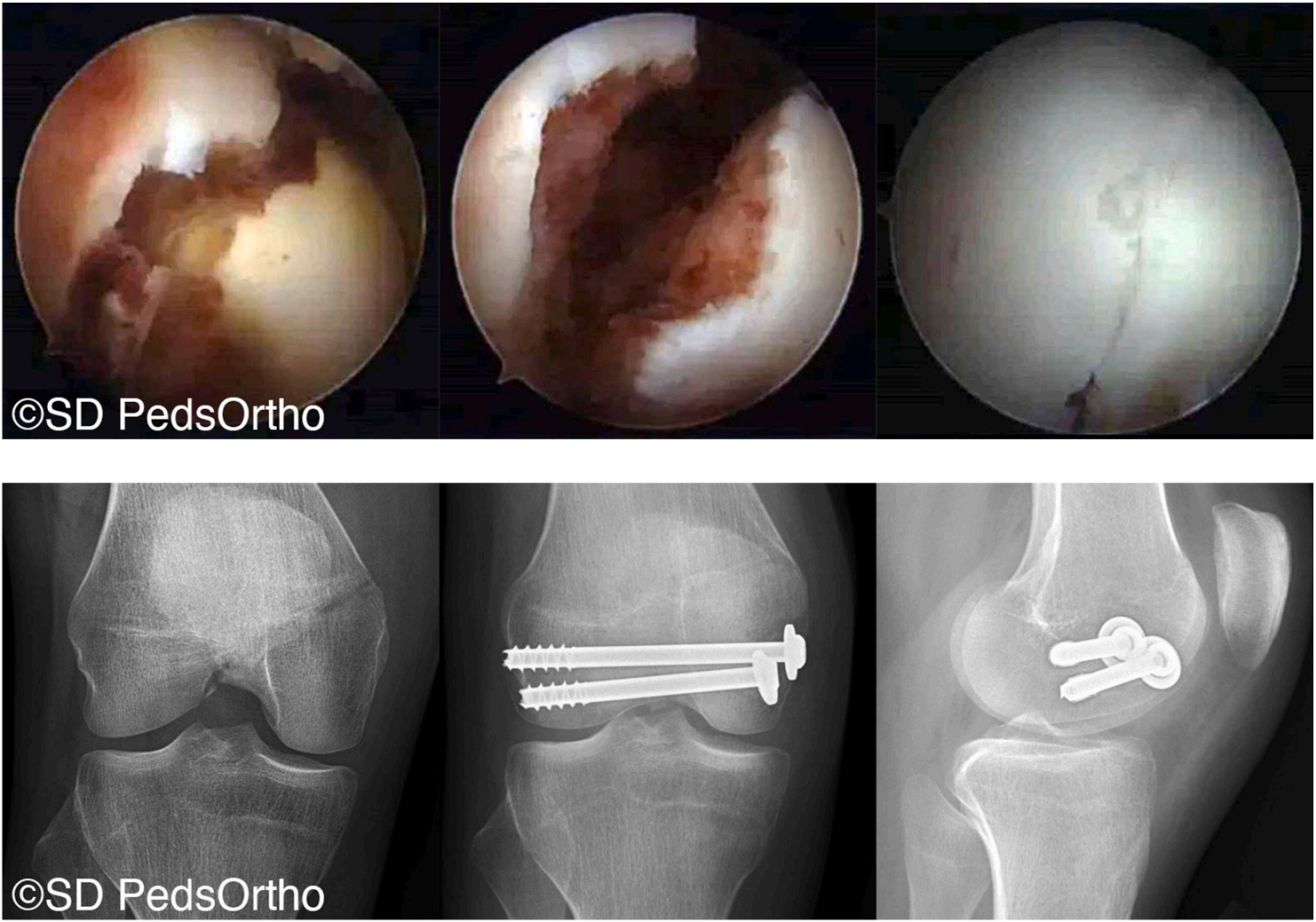
Prereduction (top row left and center image) and post-reduction (top row right image) arthroscopic images and pre- and postoperative radiographs (bottom row).

**Figure 13:**
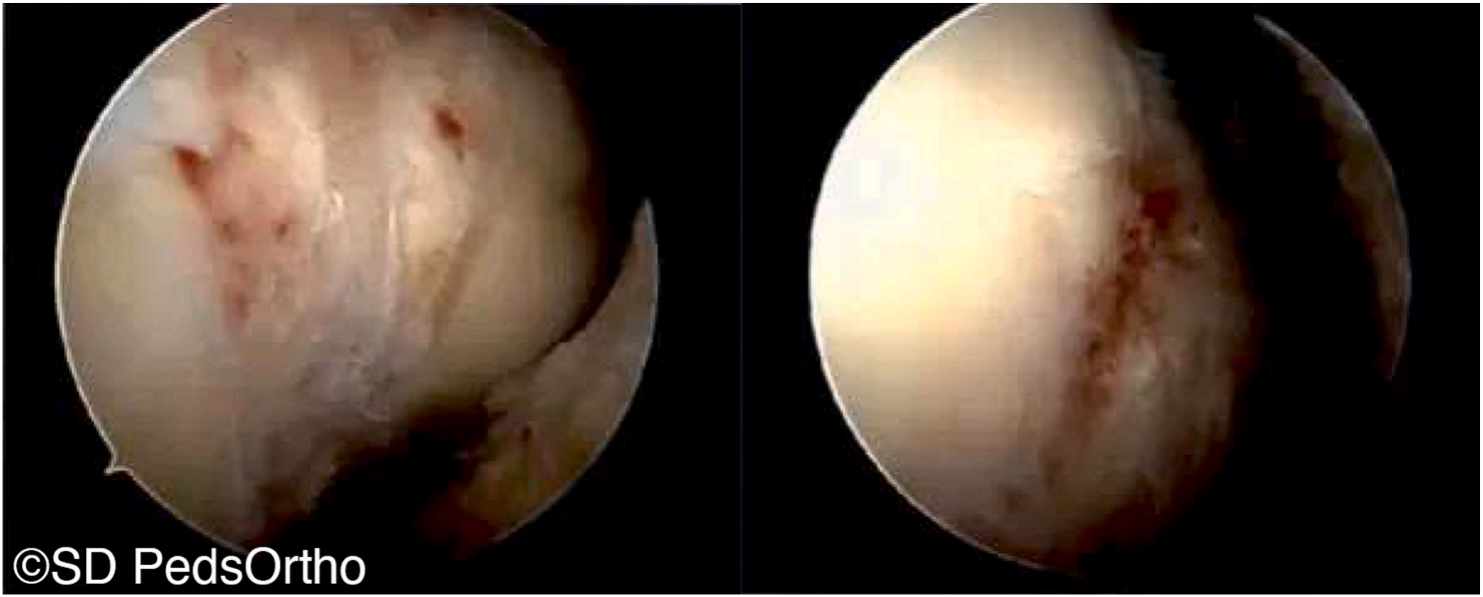
Six months following fracture fixation, adhesions to the cartilage/fracture were removed (left image demonstrating the healed fracture above the notch, and the right image demonstrating the healed fracture across the condyle after lysis of adhesions).

Case 4: A 16-year-old female sustained a right knee injury after a direct blow to the lateral knee during a rugby game, with immediate pain and a knee effusion. Two weeks later, she presented to the clinic with reports of occasional clicking and locking with knee range of motion. She then underwent an arthroscopic procedure to guide the external rotation and varus reduction maneuver. Again, an anatomic reduction was obtained, and 6.5 mm cannulated screws were used to secure the fracture (Fig. 12). She was immobilized with a knee immobilizer, but she experienced a one-month delay in starting her physical therapy. At 6 months postoperatively, she had achieved only a knee range of motion of 5–90° with physical therapy, and therefore she underwent arthroscopic lysis of adhesions to improve her motion (Fig. 13). One month later, she returned to full unrestricted sport.

## Comparison to other methods

While closed traction-based reduction is often attempted, its success is limited by rotational deformity and persistent translation. Open reduction remains the most common recommendation for displaced SH-III fractures (of any joint) [9]. Although effective, this historical recommendation carries a risk of physeal injury and increased operative morbidity. The technique described here offers targeted correction based on predictable displacement vectors from soft tissue attachments and the juxtaposition of the intact medial meniscus to the condyle, guiding the condyle into the anatomic position. This technique enhances control over rotational and translational deformity and may also reduce the need for extensive open approaches, further respecting the soft tissue envelope.

## Summary

This technical tip outlines a reproducible reduction technique for SH-III medial femoral condyle fractures. When an external rotation maneuver is combined with a varus force, accurate reduction is achieved with minimal soft-tissue disruption, thereby reducing reliance on open approaches. Other complications unrelated to the reduction or the physis are not necessarily minimized, as demonstrated by arthrofibrosis in all three arthroscopic case examples. However, key advantages of this technique include preservation of periarticular soft tissues, reduced manipulation of the physis, improved rotational and translational control of the displaced epiphyseal fragment, and reproducibility.

### Limitations

There are several limitations to the described technique. First, its applicability to highly comminuted or physeal-metaphyseal transition zone fractures remains uncertain. Second, the cadaveric study was performed on an adult human knee due to the limited availability of pediatric distal femoral specimens. Consequently, this technique would benefit from further validation in pediatric models to better account for physeal and periosteal anatomy. To mitigate this limitation, we have provided clinical case examples of patients aged 10 to 16 years, alongside an intraoperative video demonstrating the technique in a pediatric patient. Despite these limitations, this maneuver provides an effective and reproducible intraoperative technique for orthopaedic surgeons managing SH-III medial femoral condyle fractures.

## Additional links

• ChildrensOrtho: Donor Knee SH-III distal femur fracture reduction technique
• ChildrensOrtho: Open Approach intra-operative SH-III distal femur fracture reduction technique
• AO Foundation Surgery Reference Technique: open reduction and internal fixation of Salter Harris III distal femur fractures
• POSNAcademy: Distal Femur Physeal Injuries

## Author contributions

**Aaron Tran:** Writing – review & editing, Writing – original draft, Visualization, Project administration, Methodology, Investigation, Funding acquisition, Formal analysis, Data curation, Conceptualization. **Stephanie T. Kha:** Writing – review & editing, Writing – original draft, Visualization, Methodology, Investigation, Formal analysis, Data curation, Conceptualization. **Christine L. Farnsworth:** Writing – review & editing, Writing – original draft, Supervision, Resources, Project administration, Methodology, Investigation, Formal analysis, Data curation. **Eric W. Edmonds:** Writing – review & editing, Writing – original draft, Supervision, Resources, Project administration, Methodology, Investigation, Data curation, Conceptualization. **Christopher D. Souder:** Writing – review & editing, Writing – original draft, Visualization, Validation, Supervision, Resources, Project administration, Methodology, Investigation, Formal analysis, Data curation, Conceptualization.

## Ethics approval and consent

Images were collected under a retrospective institutional review board protocol; written patient consent is not required.

## Declaration of competing interests

The authors declare the following financial interests/personal relationships which may be considered as potential competing interests: C.S. reports a relationship with POSNA QSVI Trauma Committee that includes board membership and a relationship with OrthoPediatrics Corp that includes consulting or advisory and speaking and lecture fees. E.E. reports a relationship with Arthrex Inc that includes speaking and lecture fees and a relationship with the Pediatric Orthopaedic Society of North America and the Pediatric Research in Sports Medicine society that includes board membership. The other authors declare that they have no known competing financial interests or personal relationships that could have appeared to influence the work reported in this paper.

## References

[1] Siow H.M., Cameron D.B., Ganley T.J. (2008). Acute knee injuries in skeletally immature athletes. Phys Med Rehabilitation Clin North Am.

[2] MacDonald J., Rodenberg R., Sweeney E. (2021). Acute knee injuries in children and adolescents. J Am Med Assoc Pediatr.

[3] Torg J.S., Pavlov H., Morris V.B. (1981). Salter-Harris type-III fracture of the medial femoral condyle occurring in the adolescent athlete. J Bone Joint Surg.

[4] Basener C.J., Mehlman C.T., DiPasquale T.G. (2009). Growth disturbance after distal femoral growth plate fractures in children; A meta-analysis. J Orthop Trauma.

[5] Moran M., Macnicol M.F. (2006). (ii) paediatric epiphyseal fractures around the knee. Curr Orthop.

[6] Hulbert M.A., Chun L., Bryan T.P., Souder C.D., Pennock A.T. (2025). Factors associated with premature physeal closure after distal femur physeal fracture. J Pediatr Orthop.

[7] Wall E.J., May M.M. (2012). Growth plate fractures of the distal femur. J Pediatr Orthop.

[8] Lippert W.C., Owens R.F., Wall E.J. (2010). Salter-Harris type III fractures of the distal femur. J Pediatr Orthop.

[9] Levine R.H., Thomas A., Nezwek T.A., Waseem M. (Aug 10, 2023).

