# Corrigendum to “An Anatomic Reduction Technique for Salter-Harris Type III Medial Femoral Condyle Fractures” [J Pediatr Orthop Soc North Am 16 (2026) 100422]

**DOI:** 10.1016/j.jposna.2026.100445

**Published:** 2026-09-18

**Authors:** Aaron Tran, Stephanie T. Kha, Christine L. Farnsworth, Eric W. Edmonds, Christopher D. Souder

**Affiliations:** 1 Department of Orthopaedic Surgery, University of California San Diego Health, San Diego, CA, USA; 2 Division of Orthopediatrics, Rady Children's Hospital, San Diego, CA, USA

The authors regret omitting the following text from the Acknowledgements:

“The authors wish to thank individuals who donate their bodies and tissues for the advancement of education and research.”

The authors also regret including the following text in the Acknowledgements:

“Donor knee was provided via a grant from the University of California, San Diego Anatomical Program (A. Tran).”

The authors would like to apologize for any inconvenience caused.

